# QuickStats

**Published:** 2013-06-28

**Authors:** Patricia F. Adams, Michael E. Martinez

**Figure:**
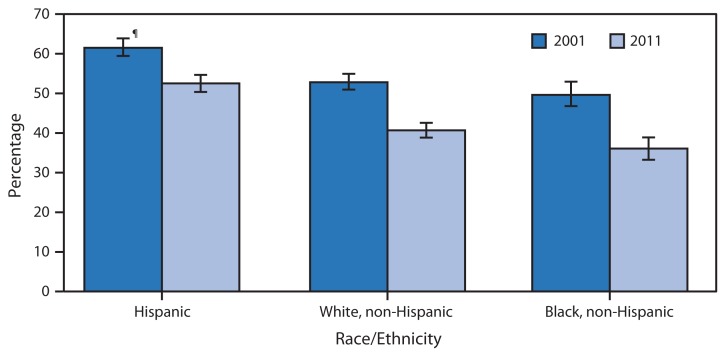
Percentage of Uninsured Persons Aged <65 Years with No Health Insurance Coverage Because of Cost,^*^ by Race/Ethnicity^†^ — National Health Interview Survey, United States, 2001 and 2011^§^ ^*^Based on the family respondent’s response to a survey question that asked about uninsured family members, “Which of these are reasons [person] stopped being covered or does not have health insurance?” Reasons included lost job or change in employment, change in marital status or death of a parent, ineligible because of age or left school, employer didn’t offer or insurance company refused, cost, Medicaid stopped, and other reason. More than one reason could be provided. ^†^Persons of Hispanic ethnicity might be of any race or combination of races. ^§^Estimates are based on household interviews of a sample of the civilian noninstitutionalized U.S. population and are derived from the National Health Interview Survey Family Core component. ^¶^95% confidence interval.

From 2001 to 2011, the percentage of uninsured persons aged <65 years for whom cost was a reason for not having health insurance coverage decreased among uninsured Hispanic, non-Hispanic white, and non-Hispanic black persons. In 2001 and 2011, uninsured Hispanic persons aged <65 years were more likely than uninsured non-Hispanic white and non-Hispanic black persons to lack health insurance coverage because of cost.

**Sources:** Barnes PM, Adams PF, Schiller JS. Summary health statistics for the U.S. population: National Health Interview Survey, 2001. Vital Health Stat 2003;10(217). Available at http://www.cdc.gov/nchs/data/series/sr_10/sr10_217.pdf.

Adams PF, Kirzinger WK, Martinez ME. Summary health statistics for the U.S. population: National Health Interview Survey, 2011. Vital Health Stat 2012;10(255). Available at http://www.cdc.gov/nchs/data/series/sr_10/sr10_255.pdf.

